# A Practical Treatise on Dental Medicine, Being a Compendium of Medical Science, as Connected with the Study of Dental Surgery, &c. &c.

**Published:** 1852-12

**Authors:** 


*A Practical Treatise on Dental Medicine, being a Compendium of Medical Science, as connected with the study of Dental Surgery*, &c. &c. *Second Edition.* Revised, corrected and enlarged, by Thos. E. Bond, A. M., M. D., Professor of Special Pathology, &c. &c. in Baltimore Dental College. Philadelphia: Lindsay & Blakiston, 1852.

Having noticed with sincere and earnest commendation, in the Medical Examiner for March, 1851, the first edition of this valuable book, we should perhaps be forgiven if disposed rather to congratulate ourselves than the gifted author, upon the appearance of the second and enlarged edition just handed us for examination. To him the demand for this extension of his work is but the fair and just result of the merit that belongs to his original labor, the successful performance of a task that brings its due reward; while to his friends it is practically a public endorsement of opinion that confirms their high estimate of the work.

The present edition, besides some minor or mechanical improvements, possesses a most sensible chapter on anæsthetics, wherein the able pen of Professor Bond has, it may be hoped, put to rest all doubts that may have existed in some minds, as to the safety or propriety, in the hands of the dentist, of these powerful agents.

The Professor says: "Painful experience has shown that the use of anæsthetics has been more dangerous in the hands of dentists than of other administrators, death having more frequently occurred in attempts to save the patient from the pain caused by the little operation of extracting a tooth, than perhaps from all the instances in which anæsthetics have been administered to prevent the consciousness of suffering in capital operations."

Since the introduction of sulphuric ether and chloroform into medical practice, and their supposed applicability to the practice of dental surgery, it has been our fortune to occupy the position of a witness, and not that of a party to their administration. Questioning their safety, and unwilling to take the responsibility of suspending the consciousness of those who needed operations, the pain of which we knew, from daily experience, could be readily endured without such intervention, it has been our invariable rule to oppose the recourse to that means of escaping one evil by risking the encounter of another, and perchance a worse. But where advice and reasoning would not satisfy the patient, we have insisted that the family physician should be in attendance and administer the stupefying vapor. In the course of perhaps fifty or sixty opportunities thus afforded for judging of the value and effects of anæsthetics in the dentist's practice, we have been confirmed in our opposition to its use. Our testimony would go to prove that, in a few cases, the patients have professed to feel no pain, or to remember none; but even in these best or successful instances, there were at times contradictory statements making the testimony of obtunded faculties, or faculties just recovering from that state, of doubtful value as to the question of the real amount and character of the pain endured from the operation. In other cases, it was as difficult a task for the physician to induce the patient to take the ether or chloroform, as it had been to obtain submission to the pain of extracting the tooth; and in several, the effects upon the stomach and nervous systems were such as to preclude the possibility of operating altogether.

With the necessity of giving in such testimony, as the result of our own experience, it will not be wondered that we hail with pleasure the direct and unanswerable condemnation from Professor Bond of the use of anæsthetics by the dentist, to avoid the pain of his operations; and we ask leave to quote one more passage from the same high authority:

"The greater danger of anæsthesia in dental operations is easily accounted for. The operation has to be performed upon an organ concealed in the cavity of the mouth. The preliminary stages of the operation, adjusting instruments, &c. cannot be accomplished until the patient has been made unconscious. The mouth then is generally found spasmodically closed, and cannot be forced open without considerable effort. In the mean time, the patient cannot continue the inhalation, the soporific effects of the anæsthetic pass off, and by the time the operator has accomplished the separation of the gum, consciousness has returned.

"In an operation upon any other part of the body than the organs through which inhalation is accomplished, the surgeon having the patient in a proper position for the immediate operation, can commence it as soon as anæsthesia is effected, and can proceed without interrupting the inhalation, which can be continued according to circumstances, the patient not being profoundly, but continually narcotized.

"But the dental operator must carry anæsthesia so far as to produce relaxation of the muscles of the jaw; or what I think is more commonly the case, to so completely overwhelm the consciousness as to obviate that instinctive resistance which seems to linger after volition appears to be suspended. When the patient begins to inhale the chloroform, his mind is intensely occupied with the anticipated attack upon his teeth, and his fears are concentrated upon the dread of the attack being prematurely made. By his eyes and hands he continually gives signals of consciousness as long at he can, and the last effort of volition is to clench the jaws as firmly as possible. Muscular life being less easily overcome than intellectual, the extreme anæsthetic effect of chloroform or ether has to be induced in order to overcome the difficulty.

"Again, the dentist cannot continue the administration of the anæsthetic while operating, at least not with any regularity; he is obliged to carry the anæsthesia so far as .o permit a certain degree of it to pass off without the restoration of consciousness. In other words, he must produce *super-anæsthesia*, because he must provide against the known evanescence of the condition."

Nor is it less reasonable to conclude that, in a difficult or protracted operation, the manipulations of the dentist would be most unfortunately affected by the constant fears in his own conscious mind, that the submission and tranquillity of his patient will cease the very next moment.

Professor Bond's excellent book should be carefully read by every dentist who has his own success and the welfare of his patients at heart. E. B. G.

